# Association between Carotid Artery Stenosis and Cognitive Impairment in Stroke Patients: A Cross-Sectional Study

**DOI:** 10.1371/journal.pone.0146890

**Published:** 2016-01-11

**Authors:** Wei Yue, Anxin Wang, Runxiu Zhu, Zhongrui Yan, Shouhuan Zheng, Jingwei Wang, Jia Huo, Yunlin Liu, Xin Li, Yong Ji

**Affiliations:** 1 Department of Neurology, Second Hospital of Tianjin Medical University, Tianjin, PR China; 2 Department of Neurology, Tianjin Huanhu Hospital, Tianjin, PR China; 3 Department of Neurology, Beijing Tiantan Hospital, Capital Medical University, Beijing, China; 4 China National Clinical Research Center for Neurological Diseases, Beijing, China; 5 Center of Stroke, Beijing Institute for Brain Disorders, Beijing, China; 6 Beijing Key Laboratory of Translational Medicine for Cerebrovascular Disease, Beijing, China; 7 Department of Epidemiology and Health Statistics, School of Public Health, Capital Medical University, Beijing, China; 8 Department of Neurology, Inner Mongolia People’s Hospital, Inner Mongolia, Hohhot, China; 9 Department of Neurology, Jining No.1 People’s Hospital, Jining, Shandong, China; 10 Department of Neurology, Yanbian University Hospital, Yanji, Jilin Province, China; 11 Department of Neurology, Harbin No. 2 Hospital, Harbin, Heilongjiang, China; 12 Department of Neurology, Yili Friendship Hospital, Yili, Xinjiang Provine, China; 13 Department of Neurology, Taian City Central Hospital, Taian, Shandong Province, China; Banner Alzheimer's Institute, UNITED STATES

## Abstract

To investigate potential associations between carotid artery stenosis and cognitive impairment among patients with acute ischemic stroke and to provide important clinical implications. We measured the degree of carotid artery stenosis and recorded the Mini-Mental State Examination score (MMSE) at admission in 3116 acute ischemic stroke patients. The association between carotid stenosis and cognitive impairment assessed by MMSE was tested using multivariate regression analysis. Other clinical variables of interest were also studied. After adjusting for age, gender, education level, marriage, alcohol use, tobacco use, physical activity, hypertension, diabetes, hypercholesterolemia, atrial fibrillation, myocardial infarction and NIHSS (National Institutes of Health Stroke Scale) score, we found that participants with high-grade stenosis of the carotid artery had a higher likelihood of cognitive impairment compared to those without carotid artery stenosis (OR = 1.49, 95%CI: 1.05–2.11, p<0.001). Left common carotid artery stenosis was associated with cognitive impairment in the univariate analysis, although this effect did not persist after adjustment for the NIHSS score. Cognitive impairment was associated with high-grade stenosis of the right carotid artery.

## Introduction

Cognitive impairment is an important public health problem. The worldwide incidence of Alzheimer’s disease is estimated to be >35 million cases, and the total estimated global costs of dementia amounted to US$604 billion in 2010 [1]. Cardiovascular risk factors have been suggested to play important roles in cognitive decline and dementias including Alzheimer’s disease [2–7]. However, the identification of effective cardiovascular biomarkers and the selection of instruments to measure cognitive function and initial cognitive decline remain controversial.

Studies investigating the association between carotid atherosclerosis and cognitive impairment have been performed in Western countries [8–11] and have demonstrated that carotid atherosclerosis may be a potential risk factor for cognitive impairment in elderly people. Carotid stenosis and carotid intima-media thickness (IMT) reflect different stages and severities of the atherosclerotic process. For instance, carotid IMT scores have been shown to be associated with cognitive decline in stroke-free individuals [12–19]. Recent population-based studies have also suggested that asymptomatic carotid stenosis is related to poorer neuropsychological performance [9, 10, 20] even with mild degrees of stenosis [10]. Prior epidemiological studies have indicated associations between carotid IMT, stenosis and cognitive decline in stroke-free individuals [8–11]. However, the results of recent studies have not been consistent in these associations [13, 20–25], and few large multi-center studies have been performed.

The aim of our population-based cohort study was to investigate the association of carotid stenosis, as measured by carotid ultrasound, with cognitive impairment in a large multi-center sample of stroke patients.

## Methods

### Study Design and Population

The present cohort was obtained from the Study on Oxidative Stress in Patients with Acute Ischemic Stroke (SOS-Stroke), a prospective, multi-center registry. The SOS-Stroke study consisted of consecutively selected patients (n = 4164) with acute ischemic stroke. Patients (age range, 18 to 96 years) who had suffered a stroke and were admitted to one of the 43 designated hospitals in China within 7 days were included in this study from January to October 2014. The inclusion criteria for SOS-Stroke were as follows: (1) patient age over 18 years; (2) neurologist-diagnosed acute ischemic stroke that was confirmed with computed tomography (CT) or magnetic resonance imaging (MRI); (3) time from initial stroke to diagnosis less than two weeks; and (4) patient-provided informed consent. The exclusion criteria were as follows: (1) bleeding or other pathological brain diseases, such as vascular malformations, tumors, abscesses, multiple sclerosis or other common non-ischemic cerebral diseases revealed via head CT and/or MRI; (2) transient ischemic attack (TIA); and (3) iatrogenic stroke due to angioplasty or surgical operation. We excluded 417 participants who had incomplete MMSE data and 631 participants who had incomplete carotid stenosis data. Finally, only 3,116 participants (2,031 men, 1,085 women) remained for analysis. The study was sponsored by the Stroke Screening and Prevention Engineering Office of the National Health and Family Planning Commission and was approved by the Ethics Committee of Beijing Tiantan Hospital, Xuanwu Hospital Capital Medical University and Peking Union Medical College Hospital, in compliance with the Declaration of Helsinki. All patients provided written informed consent prior to participation.

### Biometric Indicators

Body mass index (BMI) was calculated as the body weight (kg) divided by the square of height (m^2^). Blood pressure was measured using a mercury sphygmomanometer with a cuff of the appropriate size [26]. A 10-s 12-lead electrocardiogram was performed to evaluate the rate and rhythm of the heart after the individual had rested in the supine position for 5 min.

Blood samples were drawn by trained phlebotomists from the subjects after overnight fasting. Total cholesterol (TC) was measured using the endpoint test method. High-density lipoprotein cholesterol (HDL), and low-density lipoprotein (LDL) levels were measured using a direct test method (inter-assay coefficient of variation: <10%; Mind Bioengineering Co. Ltd, Shanghai, China). Oxidized low-density lipoprotein (oxLDL) was performed on serum samples using the ox-LDL ELISA Kit (RapidBio Lab) according to the manufacturer’s guidelines. All biochemical variables except ox-LDL were measured using an autoanalyzer (Olympus, AU400, Japan) at one of the central laboratories from 43 designated hospitals in China.

### Assessment of Potential Covariates

Information on demographic and clinical characteristics (age, sex, marital status, alcohol use, education and history of diseases) was collected via questionnaire. Marital status was divided into married and not married (including single, divorced or widowed). Alcohol use was defined as a daily intake of at least 100 ml of alcohol three days a week for more than a year. Physical activity was evaluated from responses to questions on the type and frequency of physical activity at work and during leisure time. Previous history of disease, including myocardial infarction, stroke, hypertension, diabetes, hypercholesterolemia, atrial fibrillation, and coronary artery disease, was based on the baseline examination and self-report. The use of antihypertensive, cholesterol-lowering, and glucose-lowering medications within the past two weeks before the baseline interview was also self-reported.

Hypertension was considered to be present if SBP was ≥140 mmHg, if DBP was ≥90 mmHg, or if antihypertensive medications were used [26]. Prevalent diabetes mellitus was defined as a fasting glucose of ≥7 mmol/l, a self-reported history of diabetes, or treatment with insulin or oral hypoglycemic agents for diabetes [27]. Hypercholesterolemia was defined as a history of hypercholesterolemia, total blood cholesterol levels ≥ 5.2 mmol/l, or use of cholesterol lowering medications[28].

### Ultrasound Examination

Carotid atherosclerosis was assessed by local experienced investigators using high-resolution B-mode ultrasonography with a 7.5 MHZ probe based on a slight modification of the Atherosclerosis Risk in Communities (ARIC) protocol [29, 30]. The ultrasound examination involved scanning the common carotid arteries, the carotid bifurcations, and the first 2 cm of the internal carotid arteries (ICA). The criteria for categorizing arterial stenosis were defined as follows: none; mild to moderate stenosis (1–74%); and severe stenosis (≥75%). Patients who had carotid occlusion were placed in the severe stenosis group. Unilateral and bilateral stenosis were also recorded.

### Neuropsychological Evaluation

Cognitive functioning was measured at intake using the Mini-Mental State Examination (MMSE). The MMSE is a measure of general cognitive function that measures orientation to time and place, attention and calculation, language and memory [31]. Higher scores indicate greater cognitive function, and cognitive impairment is defined as a score of less than 24 on the MMSE.

### Statistical Analysis

Statistical analyses were performed using a commercially available software program (SAS software (version 9.4; SAS Institute Inc., Cary, NC, USA)). Data were presented as the means ± standard deviations (SD) for continuous variables and as frequencies and percentages for categorical variables. We used Student’s t-test or analysis of variance (ANOVA) for non-paired samples for the comparison of normally distributed parameters and the Wilcoxon or Kruskal-Wallis test for the comparison of non-parametric variables. The Chi-squared test was applied for the comparison of categorical variables. As a third step in the analysis, the entire study population was divided into three groups according to the degree of carotid stenosis: none, mild to moderate stenosis (1–74%); and severe stenosis (≥75%). Multivariable logistic regression models were used to analysis the association between carotid atherosclerosis as a predictor assessed by carotid stenosis and cognitive impairment as the outcome assessed by the MMSE score of less than 24. We also used multivariate logistic regression models to calculate P values for the trends by treating the degree of carotid stenosis as a continuous variable. Risk factors whose P values were <0.2 in the univariate analysis and had clinical significance were selected as covariates in the multivariate models. The Hosmer-Lemeshow goodness-of-fit statistic was computed to examine the fit of the model. All variables whose univariate tests resulted in a P value ≤0.2 and had clinical significance were considered in the multivariate model. P values less than 0.05 in 2-sided tests were considered significant.

## Results

In total, 2031 men and 1085 women were registered in this study (mean age 63.42 years; range 18–96 years). Ultrasound images and neuropsychological evaluation data were obtained. Among the study population, 826 (26.51%) of patients were diagnosed as having cognitive impairment with a MMSE score less than 24.

Table 1 shows the characteristics of patients with intact cognition or cognitive impairment. The patients with cognitive impairments compared with the patients without cognitive impairments were significantly older, were more likely to be male, had a higher prevalence of atrial fibrillation, were more likely to be married, had a higher frequency of alcohol and tobacco use, were more likely to engage in physical activity, had higher frequencies of hypercholesterolemia, and had higher blood concentrations of oxLDL. Compared to patients with intact cognition, patients with cognitive impairment showed a higher frequency of high-grade carotid stenosis (10.65% versus 5.28%, p<0.001), particular in the right carotid artery (6.78% versus 3.23%, p<0.001), and an increased NIHSS (National Institutes of Health Stroke Scale) score (7 versus 3, p<0.001).

**Table 1:** Comparisons between patients with and without cognitive impairment in the carotid artery atherosclerosis study population.

Variable	Cognitively intact (MMSE ≥24) n = 2,290	Cognitively impaired (MMSE < 24) n = 826	P value
Age [mean (SD)]	62.0±11.7	67.2±11.8	<0.0001
Gender (Male)	1570 (68.6%)	461 (55.8%)	<0.0001
Married (Yes)	2157 (94.2%)	751 (90.9%)	0.0012
Alcohol use (Yes)	753 (32.9%)	207 (25.1%)	<0.0001
Tobacco use (Yes)	961 (42.0%)	258 (31.2%)	<0.0001
Physical activity (Yes)	910 (39.7%)	267 (32.3%)	0.0002
Education level			
Illiteracy/primary	2071 (90.4%)	751 (90.9%)	0.6837
Middle school or above	219 (9.6%)	75 (9.1%)	
Hypertension	1506 (65.8%)	558 (67.6%)	0.3509
Diabetes	541 (23.6%)	176 (21.3%)	0.1750
Hypercholesterolemia	283 (12.4%)	76 (9.2%)	0.0148
Atrial fibrillation	88 (3.8%)	55 (6.7%)	0.0009
Myocardial infarction	54 (2.4%)	21 (2.5%)	0.7670
Degree of carotid stenosis			
None	1857 (81.1%)	617 (74.7%)	<0.0001
1%–74%	312 (13.6%)	121 (14.6%)	
High-grade	121 (5.28%)	88 (10.65%)	
Left carotid stenosis ≥75	62 (2.71%)	42 (5.08%)	0.0029
Right carotid stenosis ≥75	74 (3.23%)	56 (6.78%)	<0.0001
NIHSS	3 (2–6)	7 (4–12)	<0.0001
oxLDL, mg/l	61.99 (44.36–69.81)	63.75 (55.05–70.57)	<0.0001

MMSE = mini-mental state examination, SD = standard deviation. NIHSS: National Institutes of Health Stroke Scale, oxLDL = oxidized low-density lipoprotein

**Fig 1:**
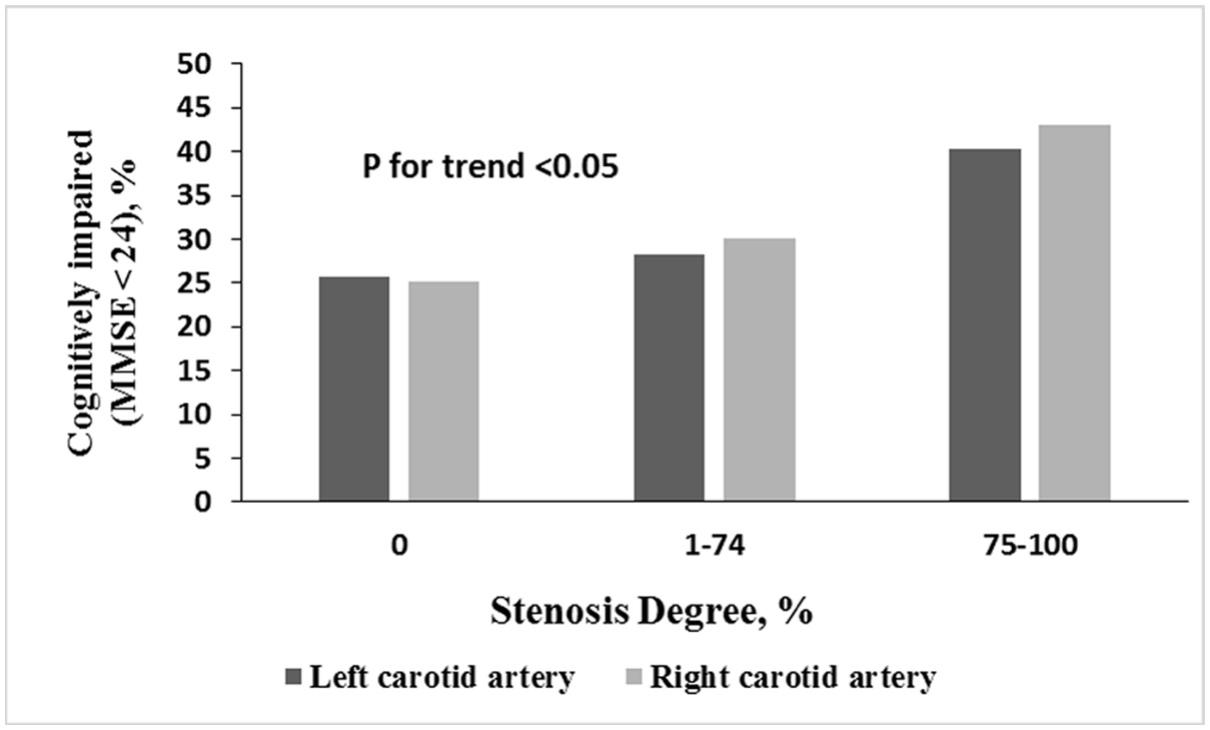
Proportions of cognitively impaired (MMSE<24) patients in the different groups according to the degree of stenosis of the left or right carotid artery.

The relationships between carotid artery stenosis and background characteristics, risk factors, and vital signs are summarized in Table 2. There were no differences in alcohol use, physical activity, education level, hypertension, diabetes mellitus, hypercholesterolemia or atrial fibrillation among patients with different degrees of carotid artery stenosis; however, age, gender, marital status, tobacco use and coronary artery disease were significantly different among patients with different degrees of carotid artery stenosis. Fig 1 shows the proportions of cognitively impaired patients (MMSE score <24) according to the level of stenosis severity and the presence of stenosis in the left and right carotid arteries.

**Table 2:** Demographic and clinical characteristics according to the degree of left and right carotid artery stenosis.

		Carotid artery stenosis							
		Left				Right			
Variable	Full cohort (n = 3166)	None (n = 2666)	1%–74% (n = 346)	High-grade (n = 104)	P value	None (n = 2637)	1%–74% (n = 349)	High-grade (n = 130)	P value
Age [mean (SD)]	63.4±12.0	62.9±12.1	66.7±11.0	65.0±10.8	<0.0001	62.9±12.1	66.4±10.5	66.1±11.8	<0.0001
Gender (Male)	2031 (65.2%)	1780 (64.1%)	245 (70.8%)	78 (75.0%)	0.0047	1695 (64.3%)	240 (68.8%)	96 (73.8%)	0.0270
Married (Yes)	2908 (93.3%)	2503 (93.9%)	306 (88.4%)	99 (95.2%)	0.0005	2470 (93.7%)	315 (90.3%)	123 (94.6%)	0.0471
Alcohol use (Yes)	960 (30.8%)	814 (30.5%)	109 (31.5%)	37 (35.6%)	0.5266	812 (30.8%)	105 (30.1%)	43 (33.1%)	0.8189
Tobacco use (Yes)	1219 (39.1%)	1002 (37.6%)	165 (47.7%)	52 (50.0%)	<0.0001	992 (37.6%)	166 (47.6%)	61 (46.9%)	0.0003
Physical activity (Yes)	1177 (37.8%)	1013 (38.0%)	132 (38.2%)	32 (30.8%)	0.3249	990 (37.5%)	133 (38.1%)	54 (41.5%)	0.6504
Education level									
Illiteracy/primary	2822 (90.6%)	2410 (90.4%)	320 (92.5%)	92 (88.5%)	0.3465	2389 (90.6%)	314 (90.0%)	119 (91.5%)	0.8646
Middle school or above	294 (9.4%)	256 (9.6%)	26 (7.5%)	12 (11.5%)		248 (9.4%)	35 (10.0%)	11 (8.5%)	
Hypertension	2064 (66.2%)	1758 (65.9%)	232 (67.0%)	74 (71.2%)	0.5140	1736 (65.8%)	242 (69.3%)	86 (66.2%)	0.4280
Diabetes	717 (23.0%)	598 (22.4%)	94 (27.2%)	25 (24.0%)	0.1392	600 (22.8%)	81 (23.2%)	36 (27.7%)	0.4243
Hypercholesterolemia	359 (11.5%)	305 (11.4%)	39 (11.3%)	15 (14.4%)	0.6385	296 (11.2%)	41 (11.8%)	22 (16.9%)	0.1377
Atrial fibrillation	143 (4.6%)	125 (4.7%)	14 (4.0%)	4 (3.8%)	0.8088	121 (95.4%)	334 (95.7%)	123 (94.6%)	0.8801
Myocardial infarction	75 (2.4%)	48 (1.8%)	21 (6.1%)	6 (5.8%)	<0.0001	54 (2.0%)	17 (4.9%)	4 (3.1%)	0.0047
NIHSS	4 (2–7)	4 (2–7)	4 (2–8)	6 (3–12)	<0.0001	4 (2–7)	4 (2–8)	5 (3–10)	<0.0001
oxLDL	62.4 (47.6–70.0)	62.4 (46.8–70.1)	62.0(48.0–69.8)	63.9 (57.1–70.3)	0.3169	62.4 (46.9–70.0)	62.5 (51.1–70.3)	63.1 (50.1–69.4)	0.5804
Cognitively impaired (MMSE < 24)	826 (26.5%)	686 (25.7%)	98 (28.3%)	42 (40.4%)	0.0029	665 (25.2%)	105 (30.1%)	56 (43.1%)	<0.0001

MMSE = mini-mental state examination, SD = standard deviation. NIHSS: National Institutes of Health Stroke Scale, oxLDL = oxidized low-density lipoprotein

Table 3 shows the results of multivariate regression analysis of the association between cognition function and carotid artery stenosis. After adjusting for potential confounders, we found that participants with high-grade stenosis of the carotid arteries had a higher likelihood of having cognitive impairment compared to those without carotid artery stenosis (OR = 1.49, 95%CI: 1.05–2.11, p<0.001). After adjustment for the NIHSS score, right carotid artery stenosis remained associated with cognitive impairment, although this effect did not persist for left carotid artery stenosis.

**Table 3:** Odds ratio of cognitive impairment by degree of carotid artery stenosis.

	Odds ratio (95% CI) by degree of carotid artery stenosis								
	Total			Left			Right		
	≥75%	1% –74%	P for trend	≥75%	1% –74%	P for trend	≥75%	1% –74%	P for trend
Case number	209	433		104	346		130	349	
Model 1 *	2.14 (1.58–2.88)	1.06 (0.84–1.34)	<0.0001	1.98 (1.32–3.00)	1.04 (0.81–1.35)	0.0088	2.19 (1.51–3.16)	1.18 (0.92–1.51)	<0.0001
Model 2 †	2.26 (1.68–3.06)	1.09 (0.86–1.38)	<0.0001	2.02 (1.34–3.6)	1.06 (0.82–1.38)	0.0055	2.34 (1.62–3.40)	1.20 (0.93–1.55)	<0.0001
Model 3 ‡	1.49 (1.05–2.11)	1.08 (1.02–1.04)	0.037	1.27 (0.79–2.04)	0.99 (0.74–1.33)	0.4846	1.66 (1.09–2.54)	1.14 (0.86–1.50)	0.020

* Adjusted for age (years), sex.

^†^Adjusted for as model 1 plus education level (elementary school, high school or college or above), marriage, alcohol use, tobacco use, physical activity, hypertension, diabetes, hypercholesterolemia, atrial fibrillation, myocardial infarction and oxidized low-density lipoprotein

^‡^ Adjusted for as model 2 plus NIHSS.

## Discussion

To the best of our knowledge, this study is the first to explore the association between carotid artery stenosis and cognitive impairment in a stroke population. In this large multi-center study of 3116 stroke patients in Chinese, we found a positive association between the degree of carotid artery stenosis, based on the stenotic artery diameter (none, 1–74%, and ≥75%), and cognitive impairment in acute ischemic stroke patients. After adjusting for potential confounders, the relationship between carotid stenosis and cognitive impairment persisted, and patients with high-grade carotid stenosis had 49% increased risk of cognitive impairment compared with those without carotid stenosis.

Previous prospective studies have suggested carotid artery stenosis to be a risk factor for cognitive decline in the general population [12, 13, 19], in the elderly population [17], and in patients with Alzheimer’s disease [14, 18]. In accordance with our results, in the Tromso study, a stroke-free subgroup of study subjects with carotid stenosis (≥35%) performed less well on cognitive tests than did subjects without stenosis [10]. Likewise, high-grade stenosis (≥75%) in the carotid artery was seen as a significant predictor of cognitive impairment in a large prospective cohort study, and the results were also significant in a subgroup with no evidence of infarction on brain MRI [9]. These findings indicating that carotid stenosis both in stroke population and stroke-free subgroup, whether mild stenosis or severe stenosis, continue to have important consequences for cognitive impairment. Some studies have indicated that decline in cognitive function was associated with cerebral hypoperfusion[32–34] caused by carotid artery stenosis. Therefore, this result highlights the potential clinical value of carotid artery stenosis as a predictor of cognitive impairment in stroke patients.

In addition, we investigated the associations between carotid artery stenosis in the left versus right carotid arteries and cognitive impairment. Interestingly, different patterns were observed between left- and right-lateralized diseases. The association between high-grade stenosis of the right carotid artery and cognitive impairment was particularly strong and persisted after adjustment for contralateral disease and risk factors for vascular disease. However, the association between high-grade stenosis of the left carotid artery and cognitive impairment was no longer significant after adjusting for NIHSS. The present study demonstrated that patients with right carotid artery stenosis had a higher prevalence of cognitive impairment, which is contrary to that reported by S. Claiborne Johnston [9]. This previous study demonstrated that patients with left carotid artery stenosis had a higher prevalence of cognitive impairment. There are two possible explanations for this discrepancy between studies. First, our study investigated the association in acute ischemic stroke patients, and the proportion of right high-grade carotid artery stenosis was higher than the proportion of left high-grade carotid artery stenosis. Second, the variability may arise from differences in the ethnicities of the study participants. Indeed, the participants in the current study were mainly Han-ethnic Chinese who had both physical and lifestyle differences in comparison to other ethnic populations.

Possible pathophysiological explanations for the relationships between carotid artery stenosis and cognitive impairment should also be considered. Arterial stenosis may decrease intracranial arterial perfusion pressure and reduce blood flow velocity, leading to hypoperfusion [32]. The decline in cognitive function caused by cerebral hypoperfusion has been termed vascular cognitive impairment [33]. The causative relationship between carotid artery disease and cognitive impairment was first proposed by Fisher in 1951, based on a necropsy case [34]. Fisher postulated that an enhancement of cerebral perfusion due to reopening ICA stenosis may have a salutary effect on cognition. Several observational studies have also suggested that cognitive function improves in some patients after endarterectomy [35–39], whereas other studies have not demonstrated such an effect [11, 40–43]. Thus, future studies of therapy for ICA stenosis should consider performing long-term follow-up to evaluate cognitive impairment in patients with right-sided disease.

Our study had several strengths. First, this study had a multi-center design based on a randomly selected population sample of patients with acute ischemic stroke from 43 hospitals throughout China. Second, multiple traditional cardiovascular disease risk factors were collected and adjusted for as potential confounders to minimize residual confounding.

However, several limitations should be noted. First, the MMSE scores can be affected by gender, age and education level [44]. However, our sample was almost entirely from an ethnic Chinese Han population, and the mean education level was higher compared with the general Chinese population. Therefore, our results may not be generalizable to the general population in China. Second, the diagnosis of diabetes was based on a single measure of fasting blood glucose and history of diabetes without detecting the 2 h postprandial blood glucose. Therefore, our study may ultimately underestimate the prevalence of diabetes mellitus. Third, this study was a cross-sectional study, which limited our ability to define a cause-effect relationship between carotid stenosis and cognitive impairment.

## Conclusion

In conclusion, cognitive impairment is a common phenomenon in stroke patients in the Chinese population. Carotid artery stenosis, especially in the right carotid artery, is correlated with cognitive impairment in stroke patients.
